# Comprehensive comparison of Bioprotect balloon and Barrigel hydrogel rectal spacers in proton therapy for prostate cancer

**DOI:** 10.1186/s12894-026-02139-9

**Published:** 2026-04-11

**Authors:** Joey Arnold

**Affiliations:** Department of Medical Physics and Treatment Planning, Oklahoma Proton Center, 5901 W Memorial Rd, Oklahoma City, OK 73142 USA

**Keywords:** Proton therapy, Rectal spacing, Prostate cancer, Barrigel, BioProtect

## Abstract

**Background:**

To compare the geometric consistency, dosimetric performance, and early clinical outcomes of the BioProtect balloon spacer versus the Barrigel hydrogel spacer in patients receiving proton therapy for prostate cancer.

**Methods:**

This retrospective study analyzed 50 prostate cancer patients treated with proton therapy: 25 with BioProtect and 25 with Barrigel. Anatomical displacement, rectal dose (V70/V60/V50), rectal wall infiltration (RWI), and early gastrointestinal (GI) toxicity were assessed. Two treatment plans per patient (prostate-only and whole-pelvis) were generated using identical optimization workflows. Geometric and dosimetric comparisons were performed using t-tests, and correlations between anatomy and dose were evaluated.

**Results:**

BioProtect achieved greater midgland (18.28 mm vs. 14.52 mm, *p* < 0.01) and apical (12.8 mm vs. 8.3 mm, *p* < 0.001) separation. Prostate-only plans with BioProtect showed significantly lower rectal V60 (2.02% vs. 6.69%) and V50 (4.28% vs. 10.16%) (*p* < 0.01). Whole-pelvis plans showed V70/V60/V50 reductions of 72.8%, 66.1%, and 66.7%, respectively (all *p* < 0.01). No RWI events occurred in the BioProtect arm, compared to three with Barrigel. Acute grade ≥ 2 GI toxicity was observed in 0% of BioProtect vs. 17.4% of Barrigel patients at 3 months.

**Conclusion:**

BioProtect demonstrated superior and more consistent rectal sparing in proton therapy compared to Barrigel. The fixed geometry balloon design yielded enhanced separation, reduced rectal dose, and fewer early GI toxicities. These findings suggest an dosimetric and clinical advantage of BioProtect in the proton therapy setting.

## Introduction

### Rationale for rectal sparing in prostate cancer radiotherapy

Prostate cancer remains one of the most commonly diagnosed malignancies worldwide, with radiation therapy serving as a definitive treatment for many patients [1]. Despite advancements in delivery techniques, including proton therapy and stereotactic body radiation therapy (SBRT), the anatomical proximity of the prostate to the rectum continues to present a persistent dosimetric challenge [2]. Rectal radiation-induced toxicity is a critical dose-limiting factor that can significantly impair long-term quality of life. A foundational review demonstrated a clear association between the rectal volume receiving ≥ 60 Gy and the incidence of Grade ≥ 2 late rectal toxicity or bleeding [2], reinforcing the importance of minimizing rectal dose to improve the therapeutic ratio.

### Proton therapy, rectal spacers, and the pursuit of clinical benefit

Proton therapy offers a distinct physical advantage via the Bragg peak phenomenon, enabling highly conformal dose deposition with minimal exit dose [3]. This property makes proton therapy particularly attractive for reducing toxicity to adjacent organs at risk, including the rectum. However, randomized evidence has not consistently demonstrated a clinical advantage for protons over photons in prostate cancer. The Phase III PARTIQoL trial reported no significant differences in gastrointestinal or genitourinary quality-of-life outcomes between patients treated with proton therapy and those receiving volumetric modulated arc therapy (VMAT) [4].

One explanation for this disconnect is variability in rectal spacer use and type. Spacers are designed to displace the rectum away from the high-dose region and are known to reduce both acute and late rectal toxicity in some settings. A recent comparative study by Dhere et al. found that rectal spacers provided greater reductions in gastrointestinal toxicity among patients treated with proton therapy than those treated with photons [5]. This suggests that the clinical benefit of proton therapy may only be fully realized when combined with effective rectal displacement. Supporting this, long-term data from Zelefsky et al. demonstrated durable reductions in rectal toxicity for patients who received both proton therapy and a spacer [6].

In contrast to prior analyses, such as Lippens et al. [7], which synthesized outcomes across multiple spacer types and predominantly photon-based studies, our investigation is focused specifically on head-to-head comparison of rectal spacers in the context of proton therapy.

### Need for direct comparative evaluation

Rectal spacers are broadly categorized into injectable hydrogels and inflatable balloon devices. Hydrogel products, such as Barrigel and SpaceOAR, are injected into the perirectal space to form a temporary physical barrier. Barrigel, composed of hyaluronic acid, allows for incremental and sculptable placement, a feature that may enable more symmetric deployment and improved dose sparing. Spacer symmetry has been shown to correlate with more effective rectal protection, as highlighted in a study by Fischer-Valuck et al., which found that asymmetric hydrogel placement can result in suboptimal rectal dose reduction and may even increase the risk of rectal wall infiltration [8]. Pinkawa et al. further demonstrated that deviations from midline symmetry significantly influence rectal dose distribution and clinical outcomes [9].

The efficacy of hyaluronic acid-based spacers has been confirmed in randomized settings. For example, Mariados et al. reported a reduction in acute Grade ≥ 2 gastrointestinal toxicity from 13.8% in controls to 2.9% in patients receiving a hyaluronic acid spacer during photon-based hypofractionated therapy [10]. These findings provide a rationale for further evaluating such devices in proton therapy, where precision and rectal sparing are even more critical.

Hydrogel spacers such as SpaceOAR (Boston Scientific) represent the most extensively studied class of injectable rectal spacers in prostate radiotherapy. In a clinical series of men undergoing hypofractionated radiotherapy, Pepe et al. (2021) reported favorable gastrointestinal outcomes and demonstrated the safety and feasibility of hydrogel spacer placement in routine practice. These findings reinforce the clinical utility of injectable spacers in reducing rectal toxicity. However, hydrogel devices remain dependent on injection technique and distribution symmetry, which may influence both geometric consistency and complication risk [11]. Given the growing adoption of proton therapy and the importance of reproducible rectal sparing, direct comparative evaluation of different spacer technologies within a proton-specific context remains warranted.

Inflatable balloon spacers, such as the BioProtect device, are surgically inserted and inflated with saline to create a fixed, symmetrical geometry, aiming to reduce operator-dependent variability. The BioProtect device, like hydrogel devices, is biodegradable and does not require any intervention after successful placement [12]. Comparative data in VMAT settings have shown promising dosimetric results. Kos et al. reported that BioProtect achieved a 17% greater reduction in rectal V70 and a 28% greater reduction in V60 compared to hydrogel-based spacers [13]. However, these findings have not been validated in proton therapy settings.

In addition to dosimetric performance, procedural safety and deployment variability are critical considerations. Rectal wall infiltration (RWI) is a known complication of hydrogel spacer insertion and has been associated with adverse events, including pain and rectal injury [8]. Registry-based analyses and manufacturer reports have raised concerns about underreported RWI incidence in real-world use, further underscoring the need to compare spacer-specific complication profiles [14].

### Study objectives

This study aims to determine the optimal rectal spacer for patients undergoing proton therapy by addressing four key objectives:

1. Geometric Comparison: To quantify the anatomical displacement achieved by Barrigel and BioProtect spacers.
2. Dosimetric Evaluation: To compare rectal dose reduction in proton treatment plans using each device.
3. Placement Assessment: To evaluate the technical success of spacer deployment and the incidence of complications such as rectal wall infiltration.
4. Early Clinical Outcomes: To assess patient-reported rectal toxicity and acute adverse events in the immediate post-treatment period.

**Fig. 1:**
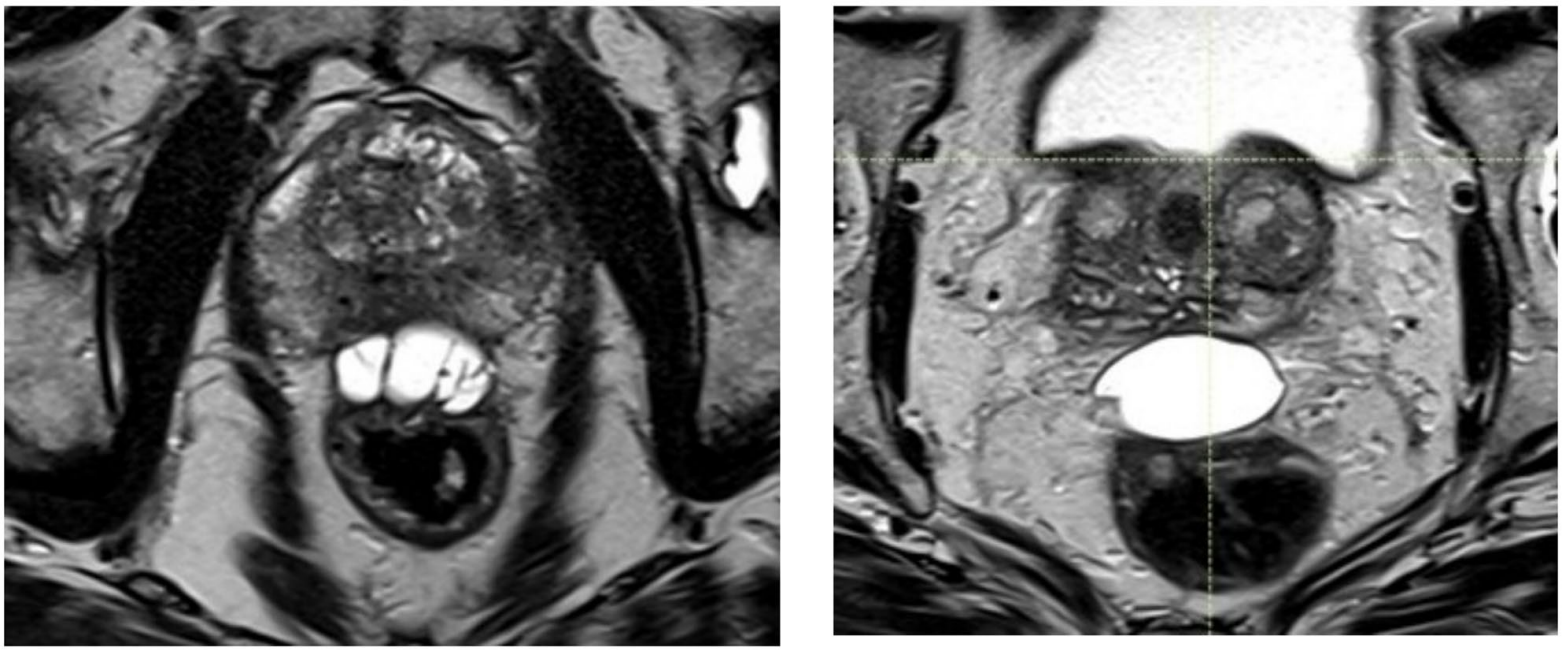
An example Barrigel placement (left), and example BioProtect placement (right)

By integrating geometric, dosimetric, procedural, and clinical outcome data, this study addresses a critical gap in the literature and provides a proton therapy-specific, head-to-head comparison of two commercially available rectal spacers. An example of the placement of each of these two devices is provided in Fig. 1.

## Methods and materials

### Study design and patient cohort

This retrospective, single-institution study included 50 prostate cancer patients treated with proton therapy and either a BioProtect balloon spacer (*n* = 25) or a Barrigel hydrogel spacer (*n* = 25). All eligible BioProtect patients treated during the study period were included. A comparator Barrigel cohort was selected chronologically, applying the same exclusion criteria to ensure a matched design.

Patients with prior prostatectomy, hip arthroplasty, or pelvic radiation were excluded. Demographic data, prostate volume, disease grade, and Gleason score were collected at baseline to characterize the cohort and ensure group comparability.

Imaging, Contouring, and Spacer Evaluation.

All patients underwent computed tomography (CT) and magnetic resonance imaging (MRI) simulation for treatment planning. Organ-at-risk and target volumes were contoured on fused CT/MRI datasets according to institutional standards. Spacer volumes were contoured on planning MRI.

Geometric evaluation included:

- Craniocaudal length and lateral width of the spacer.
- Prostate–rectum separation at mid-gland and apical levels (1 cm inferior to mid-gland).
- Minimum prostate–rectum distance, measured via isotropic expansion until intersection.
- Bladder volume within a 5-cm anterior expansion of the prostate.
- Penile bulb displacement relative to the prostate, assessed by center-of-mass shifts in three axes: anterioposterior( AP), left-right (LR), and cranio-caudal (CC).

Rectal wall infiltration (RWI) was scored by blinded reviewers using a modified ordinal scale based on T2-weighted MRI:

- Grade 0: no infiltration.
- Grade 1: discrete infiltration.
- Grade 2: <25% rectal wall circumference.
- Grade 3: ≥25% circumference.

### Treatment planning and dosimetric evaluation

Clinical treatment plans were developed to treat the prostate to 72.5GY (RBE) in 29 fractions. A hypothetical pelvis plan treating 50 Gy to the pelvic lymph nodes and prostate in 25 fractions, followed by a 30 Gy boost to the prostate in 15 fractions was also created to assess the effect of additional treatment volume on rectal spacer performance. Automated scripting workflows ensured identical optimization objectives and dose constraints to reduce inter-planner variability. This design aimed to isolate spacer type as the only variable influencing dosimetric outcomes.

The following dosimetric endpoints were extracted:

- Rectal V70, V60, and V50: the volume (%) of rectum receiving ≥ 70, ≥60, and ≥ 50 Gy, respectively.

### Statistical analysis

Statistical analysis was performed using Python. Continuous variables (e.g., V70, separation distances) were compared using two-tailed independent t-tests. Normality was verified with the Shapiro–Wilk test. For non-normal distributions, Mann–Whitney U tests were used. RWI rates were compared using Fisher’s exact test. A p-value < 0.05 was considered statistically significant.

A quartile stratification analysis was performed on anatomical separation metrics to assess the impact of spacer placement variability. Upper and lower quartiles of mid-gland separation were correlated with V70/V60/V50 to evaluate whether dosimetric superiority was preserved across placement quality.

Additionally, Pearson correlation analysis was used to identify geometric predictors of rectal dose (e.g., rectum-in-planning target volume (PTV) volume vs. V60 and V70). The strongest correlation was observed between rectal volume within the prostate PTV and rectal dose at V60/V70 levels.

### Clinical outcome assessment

Acute rectal toxicity was assessed at first on-treatment visit and at the 3-month follow-up post-radiation. Toxicities were graded using Common Terminology Criteria for Adverse Events (CTCAE) version 5.0. All toxicity data were extracted from clinical documentation and verified by the treating physician. No patients were lost to follow-up at 3 months in the BioProtect group; 23 of 25 Barrigel patients returned for evaluation.

## Results

### Patient demographics

A total of 50 patients were included in the analysis, with 25 patients in each arm (BioProtect balloon spacer and Barrigel hydrogel spacer). The mean age of the cohort was 71.4 years (range: 47–86 years), and the mean prostate volume was 54.5 cc (range: 23.9–144.3 cc), with no statistically significant difference between groups. The most common Gleason scores were 3 + 4 and 4 + 4 across both cohorts, reflecting a representative mix of intermediate- and high-risk prostate cancer.

### Anatomical geometry

The mean spacer volume was significantly greater for the BioProtect group (15.38 cc, SD 1.70) compared to Barrigel (9.98 cc, SD 1.65). Spacer length was similar between groups: BioProtect averaged 43.7 mm (SD 3.3), and Barrigel 43.9 mm (SD 7.8). However, Barrigel demonstrated a wider mean lateral width (31.4 mm, SD 5.6) than BioProtect (27.7 mm, SD 2.5).

Mid-gland separation between the prostate and rectum was significantly greater with BioProtect, measuring 18.28 mm (SD 1.28) versus 14.52 mm (SD 2.91) with Barrigel (*p* < 0.01), representing a 26% increase. Apical spacing also favored BioProtect, achieving 12.8 mm (SD 4.3) compared to 8.3 mm (SD 3.0) with Barrigel (t = 3.82, *p* < 0.001).

**Fig. 2:**
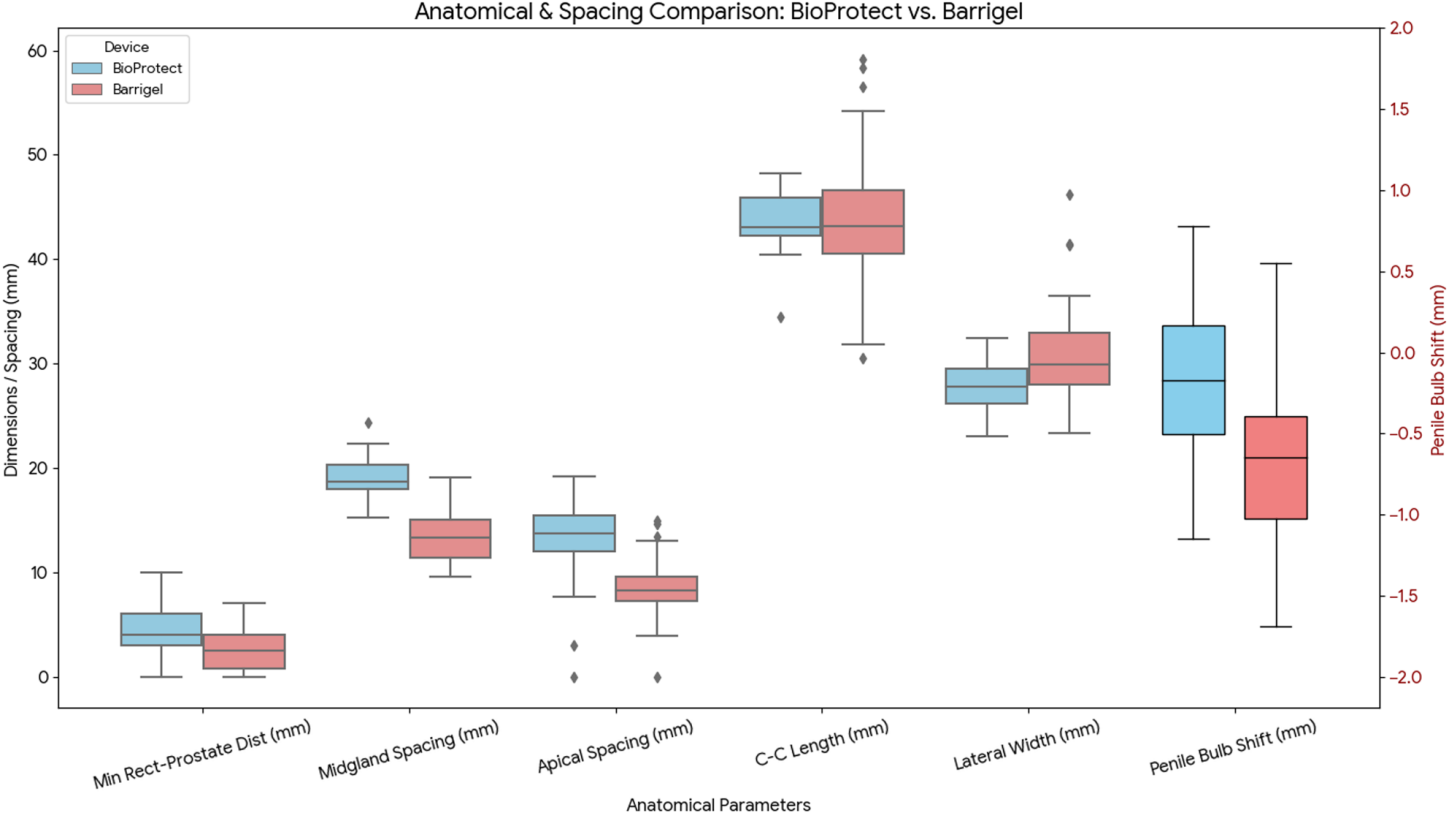
Anatomical and spacing comparison for BioProtect and Barrigel rectal spacers, including midgland spacing, apical spacing, minimum prostate–rectum distance, length and width of the spacer once it has been placed, and absolute anteroposterior shift of the penile bulb relative to the prostate

The mean minimum rectal–prostate distance was 4.64 mm (SD 2.57) for BioProtect and 2.54 mm (SD 2.21) for Barrigel. The rectal volume overlapping with the prostate PTV was markedly lower with BioProtect (0.078 cc, SD 0.193) than with Barrigel (0.498 cc, SD 0.63). The BioProtect group also demonstrated reduced anterior bladder involvement (30.49 cc, SD 15.86) compared to Barrigel (36.72 cc, SD 21.78), and a 5.2 mm posterior shift in the penile bulb–prostate relationship along the anteroposterior axis (− 0.16 cm BioProtect vs. − 0.68 cm Barrigel). No shift was observed laterally or craniocaudally. Figure 2 illustrates the results of the geometric analysis.

### Safety evaluation

Rectal wall infiltration (RWI) was absent in all BioProtect cases (0/25, 0%). In contrast, three RWI events (12.0%) were recorded in the Barrigel group: two grade 1 and one grade 2, based on MRI-based scoring. No higher-grade infiltration events were identified.

### Dosimetric comparison

Prostate-Only Plans.

For prostate-only proton plans, BioProtect showed lower rectal dose across all metrics. The mean rectal V70 was 0.14% (SD 0.39) vs. 0.55% (SD 1.05) for Barrigel; V60 was 2.02% (SD 1.97) vs. 6.69% (SD 3.23); and V50 was 4.28% (SD 3.39) vs. 10.16% (SD 4.19). These correspond to relative reductions of 74.5%, 69.8%, and 57.9%, respectively. The differences in V60 and V50 were statistically significant (*p* < 0.01); the V70 reduction approached significance (*p* = 0.1).

### Whole-pelvis plans

For whole-pelvis plans, BioProtect similarly achieved significantly lower rectal doses: V70 was 0.43% (SD 0.88) vs. 1.58% (SD 4.19); V60 was 1.60% (SD 1.58) vs. 4.72% (SD 3.08); and V50 was 3.19% (SD 2.36) vs. 9.58% (SD 4.20), with all differences reaching statistical significance (*p* < 0.01).

Among geometric parameters, the volume of rectum within the prostate PTV was the strongest predictor of rectal dose. Statistically significant correlations were observed between this volume and rectal V60 for prostate-only plans (*r* = 0.63, *p* < 0.001) and V70 for pelvic plans (*r* = 0.79, *p* < 0.001).

### Quartile analysis of spacer placement

**Fig. 3:**
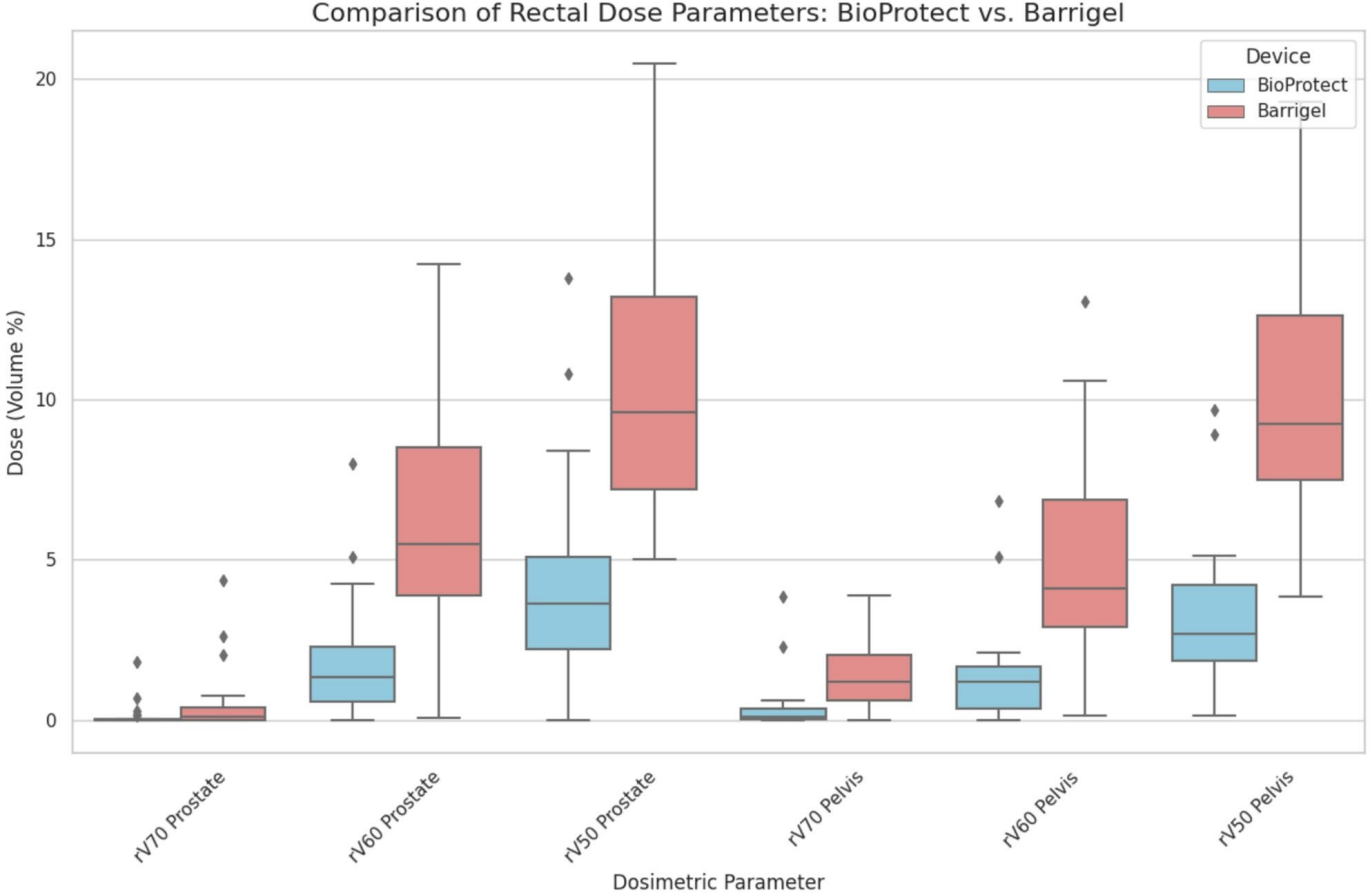
Rectal dose metrics (V70, V60, V50) are shown for prostate-only and whole-pelvis proton therapy plans

To assess the influence of spacer placement quality, a quartile-based analysis was conducted. BioProtect consistently outperformed Barrigel in both upper (optimal) and lower (suboptimal) quartiles of anatomical separation. Upper quartile prostate plan comparison resulted in BioProtect V70/V60/V50: 0.03%, 1.59%, 3.90% vs. Barrigel V70/V60/V50: 0.24%, 5.68%, 9.83%. Lower quartile prostate plan comparison resulted in BioProtect V70/V60/V50: 0.02%, 0.99%, 2.38% vs. Barrigel V70/V60/V50: 0.54%, 5.97%, 11.25%. In both quartile groups, rectal dose reductions remained statistically significant (*p* < 0.01), highlighting the dosimetric robustness of the BioProtect spacer even in less favorable placements (Fig. 3).

### Clinical outcomes

At the first on-treatment assessment, no acute rectal toxicity was reported in the BioProtect group, while three Barrigel patients presented with grade 1–2 toxicity. At three-month follow-up, no patients in the BioProtect cohort experienced grade ≥ 2 gastrointestinal (GI) toxicity. In contrast, four of 23 returning Barrigel patients (17.4%) reported grade ≥ 2 acute GI toxicities, consistent with the observed differences in rectal dose and RWI. Table 1 outlines the results of these analyses.

**Table 1:** Geometric, dosimetric, and clinical outcome analysis

Metric	BioProtect Mean (SD)	Barrigel Mean (SD)	Dosimetric Reduction (%)	*p*-value
Spacer Volume (cc)	15.38 (1.70)	9.98 (1.65)		< 0.01
Craniocaudal Length (mm)	43.7 (3.3)	43.9 (7.8)		0.94
Lateral Width (mm)	27.7 (2.5)	31.4 (5.6)		0.01
Midgland Separation (mm)	18.28 (1.28)	14.52 (2.91)		< 0.01
Apical Separation (mm)	12.8 (4.3)	8.3 (3.0)		< 0.001
Min Rectum-Prostate Distance (mm)	4.64 (2.57)	2.54 (2.21)		< 0.01
Rectum in PTV (cc)	0.078 (0.193)	0.498 (0.63)		< 0.01
Bladder Volume Anterior to Prostate (cc)	30.49 (15.86)	36.72 (21.78)		0.23
Penile Bulb Displacement (AP, mm)	0.16 cm	0.68 cm		< 0.01
RWI Events (n)	0	3		
Grade-2 GI Toxicity at 3mo (n)	0	4		
Prostate Plan				
V70 (%)	0.14 (0.39)	0.55 (1.05)	74.50%	0.1
V60 (%)	2.02 (1.97)	6.69 (3.23)	69.80%	< 0.01
V50 (%)	4.28 (3.39)	10.16 (4.19)	57.90%	< 0.01
Pelvis Plan				
V70 (%)	0.43 (0.88)	1.58 (4.19)	72.80%	< 0.01
V60 (%)	1.60 (1.58)	4.72 (3.08)	66.10%	< 0.01
V50 (%)	3.19 (2.36)	9.58 (4.20)	66.70%	< 0.01
Upper Quartile Comparison				
V70 (%)	0.03 (0.06)	0.24 (0.33)	87.50%	< 0.01
V60 (%)	1.59 (1.22)	5.68 (2.01)	72.00%	< 0.01
V50 (%)	3.90 (2.41)	9.83 (3.25)	60.30%	< 0.01
Lower Quartile Comparison				
V70 (%)	0.02 (0.05)	0.54 (0.41)	96.30%	< 0.01
V60 (%)	0.99 (0.78)	5.97 (2.34)	83.40%	< 0.01
V50 (%)	2.38 (1.64)	11.25 (3.13)	78.90%	< 0.01

## Discussion

### Spacer design, placement consistency, and dosimetric outcomes

This study demonstrates a robust association between rectal spacer design, achievable anatomical separation, and rectal dosimetry in proton therapy. Using a comprehensive evaluation framework, encompassing detailed geometric characterization, prostate-only and whole-pelvis dosimetric endpoints, and early clinical outcomes, BioProtect consistently yielded greater prostate–rectum separation and more favorable rectal dose metrics than Barrigel. These benefits were observed across multiple anatomical dimensions, including spacer volume, midgland and apical spacing, and rectal volume within the planning target volume (PTV), indicating that the observed advantage is not isolated to a specific metric or planning context.

The superior dosimetric profile of BioProtect likely derives from its fixed, balloon-based geometry, which limits inter-case variability and could reduce reliance on operator technique. In contrast, injectable hydrogels such as Barrigel, though marketed for their “sculptable” properties, appear more susceptible to variability arising from anatomical constraints and procedural factors. These observations are supported by the quartile-based analysis: BioProtect maintained lower and less variable rectal doses even in the subgroup of patients with suboptimal anatomical separation. Notably, BioProtect achieved significantly greater apical spacing.

The early clinical data are directionally aligned with the geometric and dosimetric results. No cases of rectal wall infiltration or grade ≥ 2 acute gastrointestinal toxicity were observed in the BioProtect cohort. In contrast, the Barrigel cohort exhibited three rectal wall infiltration events and four cases of grade ≥ 2 acute GI toxicity at three-month follow-up. Complication rates must be interpreted cautiously given the modest sample size. Although this difference is notable descriptively, the study was not powered to detect statistically robust differences in relatively infrequent procedural complications. Apical spacing was significantly lower in patients who experienced toxicity (mean 6.2 mm vs. 11.2 mm, *p* = 0.002), reinforcing the clinical relevance of anatomical separation in this region. Collectively, this data support the conclusion that spacer geometry, particularly the reproducibility afforded by balloon-based designs, plays a critical role in achieving consistent rectal protection in proton therapy.

Sexual function outcomes were not systematically collected in this retrospective analysis and therefore could not be compared between spacer groups. Emerging literature suggests that rectal spacers may influence erectile outcomes through indirect modulation of dose to adjacent structures such as the penile bulb and neurovascular bundles. Pepe et al. (2022) evaluated erectile function following hydrogel spacer placement combined with hypofractionated radiotherapy and reported preservation of erectile function in a proportion of treated patients [15]. Oliveira et al. reported reduced penile bulb dose with hydrogel spacers [16]. In the present study, BioProtect placement resulted in measurable anterior–posterior displacement of the prostate relative to the penile bulb, suggesting potential dosimetric implications beyond rectal sparing alone. Although penile bulb dose was not formally analyzed, these anatomical findings support prospective investigation of spacer-specific effects on sexual function and non-rectum organs-at-risk in proton therapy cohorts.

### Contextualizing dosimetric benefits within broader clinical outcomes

The dosimetric advantages observed in this study are consistent with prior data comparing balloon and hydrogel spacers. A multicenter planning study previously demonstrated that BioProtect achieved superior rectal sparing compared to control in VMAT-based photon plans [12]. Additionally, recent evidence from Dhere et al. (2024) highlights a greater clinical benefit of rectal spacers in proton therapy compared to photon-based modalities, suggesting a synergistic relationship between spacer use and the physical characteristics of proton dose deposition [5].

Rectal wall infiltration occurred exclusively in the Barrigel group, which may reflect the inherent risks of injectable hydrogel placement, including poor distribution, focal injection, or off-target deposition. BioProtect’s fixed-volume deployment may reduce placement variability; however, comparative safety conclusions require larger prospective cohorts.

Device-specific effects on adjacent pelvic structures were also observed. BioProtect resulted in an anterior–posterior offset between the penile bulb and prostate and reduced the volume of bladder anterior to the prostate. This is the first study to quantify prostate displacement relative to adjacent organs-at-risk, including the penile bulb, as influenced by rectal spacer design.

### Limitations and directions for future research

This study has several limitations. As a retrospective, single-institution analysis, the findings may not be generalizable across institutions with differing patient populations, procedural experience, or planning techniques. The sample size, while sufficient to detect significant dosimetric and geometric differences, limits the power to evaluate rare toxicity events or conduct subgroup analyses. Additionally, toxicity outcomes were limited to physician-reported acute events; patient-reported outcomes and late toxicity data were not captured.

Importantly, our 3-month follow-up is too short to comprehensively assess gastrointestinal toxicity. While the dosimetric and early clinical benefits of BioProtect are compelling, longer-term follow-up is required to confirm their durability and relevance to late gastrointestinal morbidity. Multi-institutional prospective trials incorporating both physician- and patient-reported outcomes are warranted. Comparative studies stratified by both radiation modality (proton vs. photon) and spacer type (BioProtect vs. Barrigel vs. SpaceOAR) would further clarify the optimal pairing of technologies. Economic considerations may also influence spacer selection. Pricing structures vary across institutions. The strain on resources for each procedure, cost and rate of toxicity-related interventions, and resimulation incidence all contribute to cost-effectiveness. A formal cost-analysis was beyond the scope of the present study. Future investigations should also assess the cost-effectiveness, reproducibility across learning curves, and potential workflow advantages of fixed-geometry spacers, including their impact on adaptive planning and re-simulation rates.

## Conclusion

In this retrospective cohort of prostate cancer patients undergoing proton therapy, the BioProtect balloon spacer demonstrated superior geometric consistency and dosimetric performance compared to the Barrigel hydrogel spacer. BioProtect achieved a 26% greater prostate–rectum separation at the midgland and a 54% greater separation in the apical region (12.8 mm vs. 8.3 mm), a dosimetrically sensitive area of the anterior rectal wall [2]. These geometric advantages translated into substantial reductions in rectal dose, including an approximate 70% reduction in mean rectal V60 for prostate-only plans and a 73% reduction in rectal V70 for whole-pelvis plans (*p* < 0.01 for both).

The balloon’s fixed, preformed geometry appears to drive this performance by enabling consistent and predictable tissue displacement. Quartile-based analysis further confirmed the robustness of BioProtect’s dosimetric benefit, even in cases with suboptimal separation. Apical spacing emerged as a strong predictor of rectal V70 in pelvic plans (*p* = 0.001), establishing a direct link between device-specific geometry and clinically relevant dosimetric endpoints. In this cohort, no cases of rectal wall infiltration were observed in the BioProtect group, whereas three events occurred in the Barrigel cohort. However, given the limited sample size, these findings should be interpreted cautiously and confirmed in larger prospective studies.

These findings suggest that BioProtect may offer dosimetric and geometric advantages in the proton therapy setting. Prospective, multi-institutional trials incorporating long-term follow-up and patient-reported outcomes are needed to confirm whether these dosimetric and geometric advantages translate into sustained reductions in toxicity and improvements in quality of life.

## Data Availability

The datasets generated and/or analyzed during the current study are stored in a secure institutional repository and are available from the corresponding author upon reasonable request.
